# Timely access and quality of care in colorectal cancer: a population-based cohort study using administrative data

**DOI:** 10.1186/1756-0500-6-355

**Published:** 2013-09-06

**Authors:** Geoffrey Porter, Robin Urquhart, Cynthia Kendell, Jingyu Bu, Yarrow McConnell, Eva Grunfeld

**Affiliations:** 1 Cancer Outcomes Research Program, Cancer Care Nova Scotia, Halifax, Nova Scotia, Canada; 2 Queen Elizabeth II Health Sciences Centre, Halifax, Nova Scotia, Canada; 3 Department of Surgery, Dalhousie University, Halifax, Nova Scotia, Canada; 4 Department of Community Health and Epidemiology, Dalhousie University, Halifax, Nova Scotia, Canada; 5 New Brunswick Cancer Network, Government of New Brunswick, Fredericton, New Brunswick, Canada; 6 Tom Baker Cancer Centre, University of Calgary, Calgary, Alberta, Canada; 7 Ontario Institute for Cancer Research, Toronto, Ontario, Canada; 8 Department of Family and Community Medicine, University of Toronto, Toronto, Ontario, Canada

**Keywords:** Colorectal neoplasms, Quality indicators, Healthcare quality, Access, Evaluation

## Abstract

**Background:**

While efforts to improve cancer outcomes have typically focused on improving quality of care, recently, a growing emphasis has been placed on timely access to quality cancer care. This retrospective cohort study examines, at a population level, the relationship between quality and timeliness of colorectal cancer (CRC) care in a single Canadian province (Nova Scotia). Through the provincial cancer registry, we identified all residents diagnosed with invasive CRC between 2001 and 2005 that underwent a non-emergent resection. Using anonymized administrative databases that are individually linked at the patient level, we obtained clinicodemographic, diagnostic, and treatment event data. Selected charts were reviewed to ensure completeness of chemotherapy data.

Performance on six quality indicators and the percentage of patients achieving wait-time benchmarks for diagnosis, surgery, and adjuvant therapy were calculated. The relationship between quality indicators and wait-time benchmarks was examined using logistic regression.

**Results:**

Where an association was identified, patients who received ‘higher quality care’ had longer wait times. Individuals who received a complete preoperative colonoscopy were less likely to meet benchmarks for time from presentation to diagnosis and from diagnosis to surgery. Those who received an appropriate radiation oncology consultation were less likely to meet benchmarks for time from diagnosis to surgery and from surgery to adjuvant therapy.

**Conclusions:**

As governments and other organizations move forward with strategies to reduce wait times, they must also focus on how to define and monitor quality care, and consider the relationship between these two dimensions of health care. Similarly, when developing quality improvement initiatives, the impact on resource utilization and potential to create longer waits for care must be considered.

## Background

Colorectal cancer (CRC) is a highly prevalent cancer in Canada and, despite decreased mortality in recent years, it remains the third most frequent cause of cancer-related mortality for both males and females [1]. Prognosis is largely dependent on stage at diagnosis with an estimated five-year relative survival ratio of 90% for early stage (localized) CRC, and only 12% for metastatic disease [2].

Efforts to improve CRC outcomes have typically focused on improving quality of care, defined as “the degree to which health services for individuals and populations increase the likelihood of desired health outcomes and are consistent with current professional knowledge” [3]. The measurement of the delivery of quality care has typically involved assessment of adherence to clinical practice guidelines (CPGs) and, more recently, performance on CRC-specific quality indicators (QIs).

Recently, a growing emphasis has been placed on *timely access* to quality cancer care [4-7]. Internationally, organizations have proposed wait-time benchmarks for various aspects of cancer care in order to reduce wait times; most of which have not been specific to CRC [8-13]. Given that wait times often result from limited system capacity at various points along the care trajectory (i.e., “bottlenecks”), increasing the delivery of more timely care may require additional resources, or the reallocation of existing resources. As such, implementing standards to increase timeliness may affect other aspects of care delivery, such as performance on certain quality indicators.

In a previous study by McConnell et al. [14], the achievement of several CRC-specific QIs was associated with a decreased likelihood of achieving certain wait-time benchmarks in a single health district in Nova Scotia (NS), Canada. For example, patients who received pre-operative staging imaging were less likely to receive surgery within 4 weeks of diagnosis. Based on these findings, the objective of the current study was to further explore, at a population level, the relationship between quality and timeliness of CRC care in NS.

## Methods

### Cohort

NS is a Canadian province comprised of nine health districts with a population of approximately 900,000. A population-based cohort of all individuals diagnosed with invasive CRC in NS between 2001 and 2005 was identified by the NS Cancer Registry (NSCR). From this larger cohort, our study included only those individuals who had a non-emergent resection for primary CRC (n = 2282). Individuals who presented to the emergency room and were subsequently diagnosed with CRC via emergency resection were excluded from this study as their treatment did not align with usual processes involving referral, investigation, and treatment. Patients who underwent polypectomy surgery and stage IV patients with surgery after chemotherapy were also excluded.

### Data sources

Using anonymized administrative databases that were individually linked at the patient level, we obtained clinicodemographic, diagnostic, and treatment event data. Data were obtained from: the NSCR, the Canadian Institute for Health Information Discharge Abstracts Database (DAD; inpatient diagnoses and procedures performed), Medical Services Insurance Physician Services (MSIPS; provincial physicians’ billing database that includes all physician visits and procedures), and neighborhood-level national 2001 census data. The characteristics and linkage of these databases has been published previously [15]. To ensure completeness of chemotherapy data, a chart review was performed on all stage II/III rectal and stage IIB/III colon cancer patients for whom there was a medical consultation, but no indication of chemotherapy receipt in the administrative data to determine whether the patient actually received chemotherapy. Details on the chart review are reported elsewhere [16].

### Clinicodemographics

Clinicodemographic variables included age, sex, comorbidity, cancer history, tumor location and stage, rural/urban residency, and length of stay (LOS) in hospital. Comorbidity was quantified using a comorbidity score (or count) based on the list of comorbid conditions developed by Elixhauser et al. [17]. Using the ICD-9 CM and ICD-10 coding developed by Quan et al. [18], this resulted in a list of 31 comorbidities, three of which were cancer-specific (i.e., lymphoma, metastatic cancer, and solid tumor without metastasis). To obtain a measure of comorbidity independent of the patients’ current cancer diagnoses (all patients had a confirmed diagnosis), the three cancer-specific comorbidities were excluded from the list, and each patient was assigned a comorbidity score of 0 to 28. Previous cancer diagnoses were captured by the cancer history variable, which contained a count of all previous cancer diagnoses as contained in the NSCR.

All cases of CRC were staged using the Collaborative Stage (CS) Data Collection System, version 1, resulting in CS-derived AJCC TNM stage groups [19,20]. To define patient residence as rural or urban, each patient’s residential postal code at diagnosis was assigned to a census dissemination area (DA) via the Postal Code Conversion File Plus (PCCF^+^) [21], collapsed into census subdivisions, and categorized based on classifications developed by Statistics Canada [22]. Additional details on these variables are provided elsewhere [15].

### Quality indicators

Quality care was examined using a subset of the CRC-specific QIs used by McConnell et al. [14]. Indicators were included based on their presence within the literature [23-27] and on the opinion of clinician team members that these indicators could potentially impact timely access to care. Given the study objective of exploring the relationship between quality and access at a population level, QIs were also selected based on their availability in linked administrative health databases in NS. Ultimately, six CRC-specific QIs were included:

•*Complete preoperative colonoscopy:* Complete preoperative large-bowel examination by colonoscopy, identified using procedure codes within MSIPS.

•*Margin status reported:* Pathology reporting that includes margin status, dichotomously classified (i.e., yes/no). This information is collected as part of the CS Data Collection System [19] and is available from the NSCR.

•*Adequate lymph node (LN) harvest:* Pathology reporting that includes examination of at least 12 LNs, dichotomously classified (i.e., yes/no). This information is collected as part of the CS Data Collection System [19] and is available from the NSCR.

•*Peri-operative mortality:* Death occurred in-hospital or in the 30 days following resection, dichotomously classified (i.e., yes/no). This information was obtained from the NSCR and DAD.

•*Appropriate radiation oncology consultation:* A pre-operative consultation for any rectal cancer patient, or a post-operative consultation for patients who did not receive neo-adjuvant radiotherapy and had stage II or III rectal cancer. Radiotherapy data was obtained from the NSCR.

•*Appropriate medical oncology consultation:* A post-operative consultation for all stage II-IV CRC patients. The inclusion of stage II-IV CRC patients was based on the assumption that after resection, regardless of curative or palliative intent (which cannot be determined from administrative data), these patients should receive *at least* a medical oncology consultation. Chemotherapy data was obtained from the NSCR, MSIPS, and chart review.

### Wait-time intervals

Timeliness of CRC care was assessed using wait-time benchmarks. This study used aggregates of existing benchmarks [9,11,13]: 4 weeks from presentation to clinical diagnosis, 4 weeks from clinical diagnosis to surgery, 8 weeks from surgery to start of adjuvant therapy. For each interval, the following start and end points were defined:

•Presentation date was defined as the date of the earliest physician visit for signs/symptoms suggestive of CRC within 1 year prior to clinical diagnosis. MSIPS was used to identify visits and associated diagnostic codes. Diagnosis codes suggestive of CRC included those related to gastrointestinal complaints (e.g., diarrhea, anal or rectal pain, blood in stool), anemia, weight loss, fatigue, and nausea/vomiting as well as screening for malignancy of colon or rectum.

•Clinical diagnosis date was defined as the *earliest* of the diagnosis date in the NSCR, or date of a colonoscopy/sigmoidoscopy/ barium enema performed within 3 months prior to surgery.

•Surgery date was defined as the *earliest* non- emergent surgical resection as identified within the DAD and MSIPS.

•(Neo) adjuvant therapy start dates were defined as the first date (post-resection for adjuvant therapy) that a patient received either chemotherapy or radiotherapy. For patients who received neo-adjuvant radiotherapy, the interval from clinical diagnosis to surgery was adjusted by 10 weeks to account for the delivery of radiotherapy and necessary recovery time prior to surgery.

### Analysis

Descriptive statistics were generated for clinicodemographics, QIs, and time intervals. The denominator for all QIs, except those related to oncology consultation, was the entire study cohort (n = 2282). For the QIs related to appropriate medical or radiation oncology consultation, the denominator included all patients for whom a consultation would be considered appropriate, as described previously.

Multivariate logistic analyses were performed to explore which clinicodemographics and QIs were associated with meeting each of the wait time benchmarks. Only relevant QIs were included in each model (i.e., those representing activities that would have occurred in the time up to and including that captured by each interval). For each interval, the model included the following variables:

•Presentation to diagnosis: age group, sex, comorbidity count, cancer history, rural/urban residence, tumor location, and preoperative colonoscopy. Tumor stage was not included as it was not known prior to diagnosis.

•Diagnosis to surgery: age group, sex, comorbidity count, cancer history, rural/urban residence, stage, preoperative colonoscopy, and appropriate radiation oncology consultation. Tumor location was not included since separate models were run for CRC and rectal cancer only.

•Surgery to adjuvant therapy: sex, age group, comorbidity count, cancer history, rural/urban residency, LOS, preoperative colonoscopy, margin status reported, adequate LN harvest, peri-operative mortality, appropriate radiation oncology consultation, and appropriate medical oncology consultation. Only those with a tumor stage for which adjuvant therapy is recommended were included in this analysis. As such, tumor stage was not included as a covariate.

Ethical approval for this study was obtained from the Capital District Health Authority’s Research Ethics Board (CDHA-RS/2008-049).

## Results

Cohort clinicodemographics are presented in Table 1. As shown in Table 2, performance varied substantially across QIs. Notably, margin status was reported for 94.6% of the study cohort while adequate LN harvest was obtained in only 31.8%.

**Table 1:** Clinicodemographics (n = 2282)

**Characteristic**		**n**	**%**
Age	< 50 yrs	147	6.4
	50 - 69 yrs	935	41.0
	≥ 70 yrs	1200	52.6
Sex	Male	1259	55.2
	Female	1023	44.8
Comorbidity count	0-3	2185	95.8
	≥ 4	97	4.3
History of cancer	Yes	339	14.9
	No	1943	85.1
Tumor location	Right colon	865	37.9
	Left colon	579	25.4
	Rectum	818	35.9
	Colon NOS	20	0.9
Stage	I	467	20.5
	II	785	34.4
	III	712	31.2
	IV	275	12.1
	Unkown	43	1.9
Rural/urban	Rural	876	38.4
	Urban	1406	61.6
Length of stay (post-resection)	Median (days)	9	
	Inter-quartile range (days)	5	

**Table 2:** Quality indicator performance

**Quality indicator**	**n**	**% achieved**
Complete preoperative colonoscopy	2282	57.8
Margin status reported	2282	94.6
Adequate lymph node harvest (≥ 12)	2282	31.8
Peri-operative mortality	2282	2.7
Appropriate radiation oncology consultation ^1^	514	72.6
Appropriate medical oncology consultation ^2^	1772	60.9

^1^ All stage II, III rectal patients + rectal patients who had preoperative consultation.

^2^ A post-operative consultation for all stage II to IV colorectal patients.

With regard to wait-time benchmarks (Table 3), 37.1% of patients achieved the 4-week benchmark from presentation to diagnosis; 67.4% achieved the 4-week benchmark from diagnosis to surgery; and 39.2% achieved the 8-week benchmark from surgery to start of adjuvant therapy. When only rectal cancer patients were considered in the diagnosis to surgery time interval, 56.5% achieved the 4-week benchmark.

**Table 3:** Wait-time benchmark achievement

**Wait-time interval**	**n**	**Median time (days)**	**Benchmark**	**Benchmark achievement (%)**
Presentation to diagnosis^1^	1807	44	4 weeks	37.1
Diagnosis to surgery- CRC^2^	2282	19	4 weeks	67.4
Diagnosis to surgery-RC Only^2^	818	25	4 weeks	56.5
Surgery to adjuvant therapy^3^	526	66	8 weeks	39.2

^1^Presentation date not available if there were no physician visits in the year prior to diagnosis, or if no presentation codes present consistent with those that we identified.

^2^For rectal cancer patients who received neo-adjuvant radiotherapy, this interval was adjusted by subtracting 10 weeks from the total time between diagnosis to surgery to account for the delivery of radiation and subsequent recovery time prior to surgery.

^3^Contains stage II, III rectal cancer patients and stage III colon cancer patients who received adjuvant therapy. (i.e., this includes both post-operative chemotherapy and post-operative radiotherapy).

Factors associated with meeting the wait-time benchmarks are summarized in Table 4, where several associations with QIs were identified. The receipt of a preoperative colonoscopy was associated with a decreased likelihood of meeting benchmarks for presentation to diagnosis and diagnosis to surgery. The receipt of an appropriate radiation oncology consultation was associated with a decreased likelihood of meeting benchmarks for diagnosis to surgery (for CRC and for rectal cancer patients only) and surgery to adjuvant therapy. An increased LOS in hospital was also associated with a decreased likelihood of meeting the surgery to adjuvant therapy benchmark. No other associations between the achievement of wait time benchmarks and QIs were identified (data not shown).

**Table 4:** Multivariate analyses

**Wait-time interval**	**Significant factors**		**n**	**Benchmark achievement (%)**	**OR**	**95% CI**		**p**
**Presentation to diagnosis: benchmark 4 weeks (n = 1807)**	**Rural/urban**	Urban	1112	35.1	1.0			
		Rural	695	40.4	1.2	1.0	1.5	0.03
	**Sex**	Male	969	40.3	1.0			
		Female	838	33.5	0.8	0.6	0.9	0.004
	**Complete preoperative colonoscopy**	NO	746	40.1	1.0			
		YES	1061	35.1	0.8	0.7	1.0	0.04
**Diagnosis to surgery: benchmark 4 weeks (n = 2282)**	**Age group**	Overall						0.004
		> = 70	1200	66.4	1.0			
		50-69	935	69.2	1.4	1.1	1.7	0.001
		< 50	147	64.0	1.1	0.8	1.6	0.59
	**Complete preoperative colonoscopy**	NO	962	72.5	1.0			
		YES	1320	63.7	0.7	0.6	0.9	0.001
	**Stage**	Overall						<0.001
		I	467	55.9	1.0			
		II	785	70.5	2.0	1.5	2.6	<0.001
		III	712	69.0	2.1	1.6	2.8	<0.001
		IV	275	78.2	2.5	1.8	3.5	<0.001
		UNK	43	41.9	0.7	0.4	1.4	0.31
	**Appropriate radiation oncology consultation**	Overall						<0.001
		YES	373	49.6	1.0			
		NO	445	62.3	2.4	1.8	3.3	<0.001
		Not relevant (Colon)	1464	73.5	3.3	2.6	4.3	<0.001
**Diagnosis to surgery (rectal patients only): benchmark 4 weeks (n = 818)**	**Age group**	Overall						0.02
		> = 70	354	53.1	1.0			
		50-69	394	59.9	1.6	1.2	2.2	0.004
		< 50	70	54.3	1.3	0.8	2.2	0.36
	**Stage**	Overall						0.001
		I	216	52.8	1.0			
		II	215	59.1	2.3	1.5	3.8	<0.001
		III	272	55.5	2.2	1.4	3.5	0.001
		IV	91	68.1	1.8	1.1	3.1	0.03
		UNK	24	33.3	0.7	0.3	1.7	0.38
	**Appropriate radiation oncology consultation**	YES	373	49.6	1.0			
		NO	445	62.3	2.7	1.8	4.0	<0.001
**Surgery to adjuvant therapy: benchmark 8 weeks (n = 526)**	**Length of stay**		526		0.95	0.91	1.0	0.007
	**Appropriate radiation oncology consultation**	Overall						0.01
		YES	236	29.7	1.0			
		NO	27	48.2	2.6	0.3	20.7	0.36
		Not relevant (Colon)	263	46.8	1.8	1.2	2.6	0.004

Factors associated with wait-time benchmark achievement.

## Discussion

### Quality indicators

QIs are evidence-based quantitative measures of health system performance and related outcomes that are useful for documenting/monitoring the quality of care, making comparisons over time and between institutions, and supporting quality improvement [28]. In our examination of CRC-specific QIs, we found substantial variation in performance across indicators and in comparison to the literature.

QI performance was highest for margin status reported, which is expected given the importance of margin status as a prognostic factor and as a determinant for additional surgery or adjuvant therapy. Only 31.8% of the individuals in the current study had adequate LN harvest, however, this marks improved practice in NS. An audit of a single health district, conducted in the 4 years before this study, revealed that only 22.4% of patients had adequate LN harvest [29]. Subsequent knowledge translation activities, targeting surgeons and pathologists, led to an increase in adequate nodal harvest rates in the audited district and in NS [30].

Performance for the remaining QIs identified potential areas for improvement. First, a large proportion of patients did not receive a complete pre-operative colonoscopy. A complete colonoscopy is recommended to identify synchronous polyps and/or tumors that might have been undetected on radiographic investigation (e.g., barium enema) and thus might go undetected during surgical resection of the known index tumor. The presence of tumor obstruction preventing passage of the colonoscope proximally may explain some, but unlikely all, of the patients not undergoing full preoperative colonoscopy. Second, only 60.9% of CRC patients had an appropriate medical oncology consultation, and 72.6% received an appropriate radiation oncology consultation. Higher rates were reported for a single Canadian tertiary care center located in an urban setting (82% and 81% for medical and radiation oncology, respectively) [31], thus, the differences between the two studies may reflect differences in study populations.

### Wait-time intervals

Importantly, the wait-time benchmarks presented in this paper were recommended in Canada and elsewhere but not officially endorsed as policy in NS during the study period. The purpose of this paper is not to evaluate system performance based on these benchmarks, but to illustrate how various factors impact the timely delivery of care. As noted by the United Kingdom’s National Health Service [11], “Where patients wait longer, this should be because of the needs of the diagnostic process or their personal choice, not because of built-in delays in the system of care.” In our study, increased wait times were evident not only in the diagnostic period, but also throughout treatment.

Only about one-third of patients in the current study were diagnosed within 4 weeks of presentation. As discussed by McConnell et al. [14], the processes involved in diagnosis are complex, involving expertise from a variety of individuals (primary care, diagnostic imaging, surgery, pathology), often translating into increased time. In a small jurisdiction such as NS, capacity issues may cause increased wait times for appointments, and delays in the processing and reporting of laboratory and radiology results. This is exemplified by our finding that individuals who received a complete preoperative colonoscopy were less likely to meet the benchmark for time from presentation to diagnosis. Relevant to colonoscopy, capacity issues may be related to an insufficient number of surgeons, endoscopes, or available clinical space (i.e. endoscopy suites or operating room space), to perform a high volume of colonoscopies. Interestingly, rural residents were more likely to meet the presentation to diagnosis benchmark suggesting increased system capacity in the rural districts for certain processes within the presentation to diagnosis interval.

In comparison to the presentation to diagnosis interval, a greater proportion of patients met the benchmark for the diagnosis to surgery interval (for both CRC and rectal cancer). This is perhaps reflective of more clearly defined processes and the involvement of fewer clinicians during this interval. Nonetheless, CRC patients who had a complete preoperative colonoscopy were less likely to meet the benchmark, as were rectal cancer patients who received an appropriate radiation oncology consultation, highlighting the impact of the involvement of multiple (and appropriate) clinicians on wait-times. In terms of clinicodemographics, age and stage affected benchmark achievement. For both CRC and rectal cancer patients, older individuals (≥70 years) were less likely to meet the diagnosis to surgery benchmark, suggesting a more aggressive approach to treating younger individuals. Similarly, stage IV CRC patients were more likely to meet the benchmark; however, for rectal cancer patients, stage II patients were more likely to meet the benchmark.

Finally, the surgery to adjuvant therapy interval was negatively associated with LOS and receipt of an appropriate radiation oncology consultation. The effect of LOS in hospital is indicative of surgical complications, from which recovery time is required prior to beginning adjuvant therapy.

Overall, most of the analyses did not show a significant association between QI performance and achieving wait-time benchmarks. Where an association was identified, patients who received ’higher quality care’ had longer wait times. Specifically, individuals who received a complete preoperative colonoscopy waited longer for a diagnosis and for surgery, and those who had an appropriate radiation oncology consultation waited longer for both surgery and adjuvant treatment. In these instances timeliness is appropriately sacrificed to ensure each individual receives quality care, as defined by these specific quality indicators.

### Limitations

The use of administrative data allowed us to examine the relationship between access to and quality of care at a population level, however, there are a number of limitations associated with the approach. Given the nature of administrative data, we were unable to assess the effect of patient decision-making on either QI or timeliness benchmarks, which could potentially affect the quality and timeliness of the care received. For example, individuals may choose not to have a colonoscopy or an oncology consultation, or may schedule appointments around other commitments. In addition, our definition of presentation date was based on administrative codes from physician visits. Although this approach has been used in similar studies of CRC [32,33], we could not identify a presentation date for all patients. It should also be noted that due to the use of the DAD (i.e., hospital discharge data) to compute co-morbidity, our co-morbidity count is conservative and may underestimate the number of co-morbidities present. Given that population-based imaging data were unavailable, this study could only examine the proportion of patients who received a preoperative colonoscopy, not those who may have received a complete bowel examination via barium enema + flexible sigmoidoscopy.

While this was a large population-based study, the common (or uncommon) nature of some of the quality indicators (e.g. margin status) and/or the small size of some subgroups (e.g. rectal cancer patients eligible for radiotherapy consultation) may limit the power of these analyses. Finally, this study is part of a broader body of work using administrative data based on patients diagnosed between 2001 and 2005. As such, the data used in this study may not reflect current practice (i.e., rates of QI performance or benchmark achievement). However, the purpose of this study was not to evaluate cancer system performance in NS, but to demonstrate the complex conceptual relationship between measures of access and quality of care, which remains relevant in the NS context wherein resource and capacity issues are prevalent.

## Conclusion

The relationship between quality of and timely access to care is complex. For individuals with CRC, where an interdisciplinary approach to care is necessary, the various individuals and processes involved along the care continuum lead to inherent system waits. These waits may be amplified as challenges in treatment arise as a result of disease histology, patient comorbidity, or toxicity. Thus, increased wait times may be appropriate and help ensure the patient receives high quality care.

At the system level, we rely on metrics such as QIs and wait-time benchmarks to evaluate performance. Arguably, however, recent political emphasis has been on wait times and setting/achieving wait-time benchmarks rather than on monitoring processes of care that reflect quality (e.g., QIs). As governments and other organizations continue to move forward with strategies to reduce wait times, our findings suggest they must also focus on defining quality care, establishing mechanisms to identify and monitor quality care, and giving thoughtful consideration to the relationship between these two dimensions of health care. Similarly, when developing quality improvement initiatives, the impact on resource utilization and potential to create longer wait times for care must be considered. In other words, we must beware of setting standards that we do not have the system capacity to achieve.

### Consent

This study was carried out using anonymized administrative data. All data were de-identified prior to being accessed by the research team. As such, consent from individual patients was not required. All necessary institutional approvals, including Research Ethics Board approval, were obtained.

## Abbreviations

CPG: Clinical practice guideline; CRC: Colorectal cancer; CS: Collaborative staging; DAD: Discharge abstracts database; LOS: Length of stay; MSIPS: Medical services insurance physician services; NS: Nova Scotia; NSCR: Nova Scotia cancer registry; PCCF: Postal code conversion file; QI: Quality indicator.

## Competing interests

The authors declare that they have no competing interests.

## Authors’ contributions

GP and RU were involved in study conception and design and interpretation of results. CK was involved in interpretation of results and drafting the manuscript. JB performed data analysis and was involved in interpretation of results. YM and EG were involved in study conception and design. All authors read and approved the final manuscript.
